# Screen time and addictive use of gaming and social media in relation to health outcomes

**DOI:** 10.3389/fpsyg.2023.1258784

**Published:** 2023-12-18

**Authors:** Jonas Burén, Sissela B. Nutley, Lisa B. Thorell

**Affiliations:** Department of Clinical Neuroscience, Karolinska Institutet, Stockholm, Sweden

**Keywords:** internet gaming disorder, social media disorder, screen time, health outcomes, sleep

## Abstract

**Introduction:**

This study examined associations between screen time and addictive use (i.e., heavy involvement and negative consequences) of gaming and social media, and their independent effects on health outcomes.

**Methods:**

Survey data were collected from 2,265 participants (mean age = 21.57). Internet Gaming Disorder (IGD) and Social Media Disorder (SMD) were measured with the Gaming and Social Media Questionnaire (GSMQ-9), with separate measures for heavy involvement and negative consequences. Screen time was measured by weekly hours of gaming and social media. Assessed health outcomes were psychological problems, low self-concept, social problems, sleep problems, and sleep time.

**Results:**

Screen time and addictive use were significantly associated for both gaming and social media, with associations being stronger for symptoms of heavy involvement compared to symptoms of negative consequences. However, despite significant associations, a substantial proportion of the participants with a high screen time did not meet any or just one symptom of addiction. More importantly, it was primarily negative consequences that had independent effects on health outcomes, except for sleep. High levels of heavy involvement in gaming, were even related to lower, not higher, levels of psychological problems.

**Conclusion:**

The present findings study show that screen time is a poor indicator of addictive use of gaming and social media. Given that it was primarily negative consequences of gaming or social media that had effects on health outcomes, our study also emphasizes the need to distinguish between different types of addictive use and to further examine the diagnostic validity of the nine IGD symptom criteria.

## 1. Introduction

The latest version of the Diagnostic and Statistical Manual of Mental Disorders (DSM-5; American Psychiatric Association, 2013) introduced Internet Gaming Disorder (IGD) as a condition for further study. It has also been argued that excessive use of social media can be addictive, and that Social Media Disorder (SMD) can be defined using the same criteria as for IGD (e.g., van den Eijnden et al., 2016). Many studies have also found that both gaming and social media are related to negative health outcomes (e.g., Huang, 2017; Cheng et al., 2018; Hou et al., 2019; Appel et al., 2020; Twenge and Farley, 2021; Santos et al., 2023). However, results of previous studies differ regarding the strength of the effects and one important reason for this could be that some studies have focused on time spent using digital media and others on the extent to which the use is considered addictive. A relatively recent meta-analysis by Cunningham et al. (2021) concluded that it is problematic social media, not screen time for social media or intensity of social media use more generally, that is primarily linked with depression. It has also been argued that it is important to distinguish between behavior that is based on passion for gaming and behavior that is addictive or else we run the risk of over-diagnosing some individuals (e.g., Kardefelt-Winther et al., 2017; Infanti et al., 2023). However, few studies have examined different types of addictive use of digital media and screen time within the same analyses (i.e., controlled for the overlap between these constructs). The aim of the present study was therefore to examine the association between screen time and two types of addictive use (i.e., heavy involvement and negative consequences) for both gaming and social media, and whether associations are of the same strength for these two types of digital media. More importantly, the present study aimed to examine to what extent screen time, heavy involvement, and negative consequences have independent effects in relation to health outcomes.

### 1.1. Screen time and symptoms of IGD/SMD

According to the DSM-5 (American Psychiatric Association, 2013), IGD is defined by the following nine symptom criteria: (1) Preoccupation; (2) Withdrawal; (3) Tolerance; (4) Unsuccessful attempts to control; (5) Loss of interest; (6) Continued excessive use; (7) Deception; (8) Escape; and (9) Jeopardizing career/relationships. In the present study, the term addictive use refers to these nine symptom criteria, whereas screen time refers to time spent gaming or using social media. To meet the diagnostic criteria for IGD or SMD, a substantial amount of screen time is likely required (Pontes et al., 2022), and the DSM-5 (American Psychiatric Association, 2013) states that individuals with IGD typically devote 8–10 h/day or at least 30 h/week to gaming. Studies that have examined the association between screen time and addictive use have found support for this association for both gaming (e.g., Männikkö et al., 2015; Bouna-Pyrrou et al., 2018; Jeong et al., 2021; Jin et al., 2022; Jo et al., 2022) and social media (e.g., Bouna-Pyrrou et al., 2018; Guo et al., 2021; Šablatúrová et al., 2022; Tomczyk and Lizde, 2023). However, most previous studies did not measure addictive digital media use based on the nine criteria presented for IGD in the DSM-5 (American Psychiatric Association, 2013). As the validity of these nine symptom criteria have been questioned (e.g., Griffiths et al., 2016; Castro-Calvo et al., 2021), it should be considered important to investigate how each one of these symptoms are related to both screen time and negative health outcomes. To better understand the importance of specific DSM-5 symptoms, some previous studies have made a distinction between symptoms linked to heavy involvement (i.e., criteria 1 to 4 as presented above) and those associated with negative consequences (criteria 5 to 9), with the former being more strongly associated with screen time but less strongly associated with negative psychosocial outcomes (Wichstrøm et al., 2019; Burén et al., 2021, 2023). Few studies have included both social media and gaming within the same study, even though it has been argued that the DSM-5 criteria presented for IGD can also be used for SMD (e.g., van den Eijnden et al., 2016). It has therefore not been examined if the association between screen time and addictive use are similar in strength for these two types of digital media. Gaming and social media differs in terms of content and usage pattern, with individuals tending to use social media more frequently throughout the day, but in shorter sessions compared to gaming. A study by Bouna-Pyrrou et al. (2018) found stronger associations between screen time and addictive use for gaming compared to social media use, but this is an issue that needs further investigation.

As mentioned above, it is stated in the DSM-5 (American Psychiatric Association, 2013), that those meeting the symptom criteria for IGD typically spent at least 30 h/week gaming. An important type of analyses would therefore be to calculate the mean number of hours spent on gaming and social media depending on the number of symptom criteria being endorsed for either IGD or SMD. Pontes et al. (2022) observed an almost perfectly linear relation between number of endorsed symptom criteria for IGD and time spent gaming, with the mean screen time being about 35 h for males and 32 h for females for those with five IGD symptoms (i.e., the cut-off for diagnosis). However, Slack et al. (2022), who only included those playing at least 30 h/week, demonstrated that only a one-third could be classified as having IGD. Thus, we need more studies investigating how many hours that those with IGD typically spend on gaming, as well as to what extent there are individuals with a high screen time for gaming, but with low IGD symptom levels. Similar analyses should be conducted for screen time for social media use and SMD symptom levels.

### 1.2. Associations of screen time and IGD/SMD with health outcomes

Addictive use of both gaming and social media has been shown to be related to health outcomes including psychological problems, social problems, self-esteem, and sleep problems (e.g., Männikkö et al., 2015; Milani et al., 2018; von der Heiden et al., 2019; Boer et al., 2022; Zhao et al., 2022). Similar associations have also been found for screen time for gaming (Hale and Guan, 2015; Maras et al., 2015; Twenge and Farley, 2021), whereas findings are more mixed for screen time for social media. While some studies have found associations between screen time for social media and both psychological problems and stress (e.g., Twenge, 2019; Schønning et al., 2020), other studies (e.g., Coyne et al., 2020; Tang et al., 2021; Ferguson et al., 2022) found either non-significant or negligible effects on health outcomes.

The studies presented above indicate that screen time and addictive use for gaming and social media have been linked to negative health outcomes. However, as also shown above, screen time and addictive use are inter-related. It should therefore be considered important to investigate to what extent screen time is associated with negative mental health outcomes when taking the effect of addictive use into account. For instance, it is likely that individuals who engage in excessive use of digital media also exhibit symptoms of addictive use, which may contribute to negative health consequences. If this is true, we would expect the effect of screen time to disappear when controlling for addictive use and this would also indicate that it is not until the use becomes addictive that it has negative influences mental health. However, it is also possible that screen time and addictive use have additive effects on mental health (i.e., that either high screen time or addictive use has negative effects and that those with both high screen time and additive use are at highest risk). Finally, it should be noted that it is possible, or even likely that the associations vary depending on the outcome. For example, because extensive screen time crowds out health-promoting activities such as sleep or exercise (e.g., Twenge, 2019), it is possible that associations to sleep and exercise is stronger for screen time compared to additive use.

### 1.3. Aims of the present study

To address the limitations of previous studies and provide and more in-depth understanding of the association between screen time and addictive use for both gaming and social media, the present study aimed to investigate the following research questions:

1. What is the association between screen time and addictive use for gaming and social media and are the associations stronger for one type of digital media compared to the other?
2. What is the proportion of individuals with (a) a high amount of screen time (i.e., 30 h/week), but with a low number of symptoms of either IGD or SMD and (b) high number of symptoms of IGD/SMD, but with a low amount of screen time.
3. Do screen time and addictive use for gaming or social media have independent effects in relation to health outcomes when controlling for the overlap between screen time and addictive use?

## 2. Methods

### 2.1. Participants and procedure

The study included data from 2,265 participants with a mean age of 21.57 years (SD = 5.10). A total of 909 participants (40.1%) were adolescents (age 15–19 years old), and 1,356 (59.9%) were university students (age 20–39 years old). The number of females was 1,400 (60.7% for adolescents and 62.5% for university students), and the number of males were 842 (37.4% for adolescents and 37.0% for university students). In addition, 23 participants (1.9% for adolescents and 0.4% for university students) chose the option “other” when asked to identify their sex. The university students were recruited via social media, flyers, face-to-face interaction on university campuses, and through contacts with university professors. The high school students were recruited via high school teachers who distributed the link to the survey to their students. The participants were living in both urban and rural areas across Sweden. Data were collected using an anonymous online survey. The study was conducted in line with the local ethical guidelines for research on human subjects.

### 2.2. Materials

#### 2.2.1. Digital media

Screen time for gaming and social media was measured by asking the participants about their daily usage on a typical weekday and weekend day. The participants’ responses were then summed to calculate the number of hours of usage in a typical week.

IGD and SMD criteria were measured using the Gaming and Social Media Questionnaire (GSMQ-9; Burén et al., 2023), which contains the nine symptom criteria for IGD (American Psychiatric Association, 2013) and identical nine items for SMD. Each item is rated on a scale from 0 = *strongly disagree* to 4 = *strongly agree*. Factor analysis (Burén et al., 2023) has shown that this scale includes two subscales, with the subscale Heavy involvement including the first four criteria and subscale Negative consequences including the last five criteria (see Table 1 for more details). The mean values for each subscale for IGD and SMD were used in the analyses. We also calculated the number of symptom criteria for IGD and SMD. Participants were considered to meet a symptom criterion if they scored ≥3. In line with the DSM-5 (American Psychiatric Association, 2013), an individual was considered to meet the full symptom criteria for IGD if meeting ≥5 of 9 symptoms and the same cut-off was used for SMD. The psychometric properties of the GSMQ-9 has been shown to be adequate in previous research (Burén et al., 2023) and the internal consistency was adequate also within the present study for both IGD (Heavy involvement: *α* = 0.85, *ω* = 0.85; Negative consequences: *α* = 0.70, *ω* = 0.73) and SMD (Heavy involvement: *α* = 0.79, *ω* = 0.79; Negative consequences: *α* = 0.73, *ω* = 0.74).

**Table 1:** Partial correlations (controlling for age and sex) between screen time and IGD/SMD symptom severity, with each symptom criteria separated for Heavy involvement and Negative consequences.

	Screen time		
	Gaming	Social media	*z*^a^
Heavy involvement	0.70***	0.46***	12.36***
Preoccupation	0.67***	0.43***	11.72***
Withdrawal	0.53***	0.36***	7.12***
Tolerance	0.54***	0.33***	8.73***
Unsuccessful attempts to control	0.53***	0.32***	8.63***
Negative consequences	0.58***	0.35***	9.92***
Loss of interest	0.41***	0.23***	6.73***
Continued excessive use	0.46***	0.26***	7.72***
Deception	0.32***	0.19***	4.65***
Escape	0.52***	0.25***	10.72***
Jeopardizing career/relationships	0.26***	0.27***	−0.36

*z*, *z*-value from Fisher’s *r* to *z* transformation. *p < 0.05, **p < 0.01, and *p < 0.001.

a Indicates significant differences with Holm–Bonferroni correction.

#### 2.2.2. Health outcomes

The health outcomes were measured with five constructs: psychological problems, low self-concept, social problems, sleep problems, and sleep time. Psychological problems were measured with four items (i.e., irritability, nervousness, stress and feeling low) from the HBSC symptom checklist (Inchley et al., 2020). Ratings are made on a scale ranged from 1 = *Rarely or never* to 5 = *About every day*, and we used the mean score (*α* = 0.88, *ω* = 0.88), with higher values indicating higher problem levels.

In addition, we used the subscales “Low self-concept” (five items, e.g., “feeling bad about yourself”) and “Social problems” (six items, e.g., “trouble getting along with other people”) from the Weiss Functional Impairment Rating Scale (WFIRS-S; Weiss, 2000). The scale ranged from 1 = *definitely not true* to 5 = *definitely true*, and we used the mean scores (Low self-concept *α* = 0.91, *ω* = 0.91; Social problems *α* = 0.88, *ω* = 0.88) for each subscale with higher scores indicating more problems.

Sleep problems was measured with two items asking about daytime drowsiness and difficulties falling asleep. The items were assessed using a 5-point scale ranging from 1 = *Rarely or never* to 5 = *About every day*. Both items were averaged, with higher scores indicating greater sleep problems. The Pearson’s correlation between the two items was found to be 0.42. Sleep time was measured by asking the participants about the time they wake up and the time they go to sleep on a typical weekday and weekend. This information was used to calculate the average hours of sleep per day.

### 2.3. Statistical analyses

Due to the presence of outliers in the screen time variables, we applied the outlier labeling rule, multiplying the interquartile range by a factor of 2.2 to adjust the outlier scores (Hoaglin and Iglewicz, 1987). Regarding the main analyses, partial correlations (adjusting for age and sex) were first of all used to examine association between screen time and both the two SMD/IGD subscales, as well as each one of the IGD/SMD symptoms. We used age and sex as covariates because our study included a relatively broad age range and because previous research has shown that females and males differ regarding IGD/SMD symptom severity (Su et al., 2020). Fisher’s *r* to *z* transformations were used to determine whether associations between screen time and addictive use were significantly stronger for gaming compared to social media or vice versa. Second, we calculated the mean number of hours of digital media usage per group of participants meeting each number of criteria for IGD/SMD (i.e., 1–9). Third, partial correlations (adjusting for age and sex) were used to examine screen time, Heavy involvement, and Negative consequences in relation to health outcomes. Finally, hierarchical regression analyses were used to examine independent effects of screen time and the two IGD/SMD subscales, respectively. Age and sex were entered in Step 1, followed by screen time, Heavy involvement and Negative consequences in Step 2. Holm–Bonferroni corrected value of *p*s were used (Holm, 1979). The value for the Variance Inflation Factor (VIF) was <2.83 for all predictors, indicating that multicollinearity was not a problem.

## 3. Results

The average reported screen time was 12.74 h/week (SD = 10.63) for gaming and 23.43 h/week (SD = 13.59) for social media. A total of 35 participants (1.7%; 2.0% for adolescents and 1.2% for university student) met the symptom criteria for IGD, and 126 (5.6%; 5.6% for adolescents and 5.5% for adults) met the criteria for SMD.

As shown in Table 1, screen time for gaming was strongly associated with both Heavy involvement and Negative consequences. All nine IGD symptom criteria were also significantly related to screen time, with Jeopardizing career/relationships having the weakest association and preoccupation the strongest. Similarly to gaming, screen time for social media was significantly related to both Heavy involvement and Negative consequences as well as to each one of specific symptoms. Deception having the weakest association and Preoccupation the strongest. When comparing the strength of the correlations (see Table 1), associations between screen time and addictive use were significantly stronger for gaming compared to social media, except for Jeopardizing career/relationships. We also found that screen time was more strongly associated with Heavy involvement compared to Negative consequences for both gaming (*z* = 6.83, *p* < 0.001), and social media (*z* = 4.41, *p* < 0.001).

**Figure 1:**
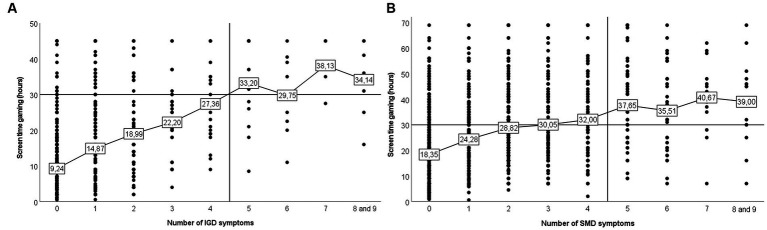
Figure showing average weekly gaming and social media screen time in relation to number of IGD/SMD symptom criteria.

Next, we calculated the mean screen time for participants meeting a specific number of symptoms for either IGD or SMD. The horizontal black line in Figures 1A,B marks the screen time cut-off of 30 h/week that is presented in the DSM-5, and the vertical black line marks the symptom cutoff for IGD/SMD (i.e., ≥5 symptom criteria). For gaming (Figure 1A), screen time increased from low levels (*M* = 9.24 h/week) for participants meeting no symptom criteria to high levels (*M* = 34.14 h/week) for participants meeting 8–9 criteria. If studying only the individuals meeting the criteria for IGD (i.e., right of the vertical line in Figure 1A), 35.3% had an average screen time below 30 h/week. Furthermore, of the participants spending at least 30 h/week on gaming (i.e., above the horizontal line in Figure 1A), 45.8% did not meet any or only one symptom criterion for IGD. Similarly to gaming, social media (Figure 1B), also showed an increase in screen time from lower levels (*M* = 18.35 h/week) among participants meeting no criteria, to high levels (39.00 h/week) for participants meeting 8–9 criteria. For those meeting the symptom criteria for SMD, 31.5% had a screen time below 30 h/week. Among participants using social media for at least 30 h/week, 48.6% did not meet any or only one symptom criterion for SMD.

Regarding associations with health outcomes, both screen time and addictive use (i.e., Heavy involvement and Negative consequences) showed significant associations in the excepted direction for all health outcomes (see Table 2). However, the associations were very small for sleep time (all *r*s ≤ 0.11). For the remaining variables, the strength of the correlations varied substantially between screen time (0.07 to 0.16), Heavy involvement (0.09 to 0.28) and Negative consequences (0.19 to 0.40). The regression analyses (Table 3) revealed that for gaming, the Negative Consequences subscale was significantly associated with all health outcomes, except for sleep time. However, for the subscale heavy involvement, higher levels were associated with *lower* health problems, although this effect was very small and only significant for psychological problems. Higher screen time was only significantly associated with reduced sleep time. In total, screen time and the two measures of addictive use of gaming accounted for between 2% (sleep time) and 13% (low self-concept) of the variance. Regarding social media, the Negative Consequences subscale was again significantly associated with all health outcomes, except for sleep time. Higher levels of Heavy Involvement with social media were significantly associated with lower levels of self-concept. Higher screen time for social media was significantly associated with more sleep problems and reduced sleep time. The explained variance ranged from 2% (sleep time) to 19% (low self-concept).

**Table 2:** Partial correlations (controlling for age and sex) examining if screen time and symptoms of heavy involvement and negative consequences for both gaming and social media are associated with health outcomes.

	Gaming			Social media		
	Screen time	Heavy involvement	Negative consequences	Screen time	Heavy involvement	Negative consequences
Health outcomes						
Psychological problems	0.07**	0.09***	0.22***	0.16***	0.22***	0.31***
Low self-concept	0.16***	0.18***	0.30***	0.16***	0.28***	0.40***
Social problems	0.16***	0.17***	0.24***	0.14***	0.21***	0.32***
Sleep problems	0.13***	0.13***	0.19***	0.15***	0.16***	0.19***
Sleep time	−0.11***	−0.08**	−0.08**	−0.10***	−0.08***	−0.08***

*p < 0.05, **p < 0.01, and ***p < 0.001.

**Table 3:** Results of hierarchical regression analyses.

	Gaming		Social media	
	*β*	*R* ^2^	*β*	*R* ^2^
Psychological problems				
Step 1:Age & sex		0.08		0.08
Step 2		0.12		0.17
Screen time	−0.05		0.05	
Heavy involvement	−0.09*		0.05	
Negative consequences	0.29*		0.27*	
Low self-concept				
Step 1:Age & sex		0.04		0.04
Step 2		0.13		0.19
Screen time	0.00		0.00	
Heavy involvement	−0.05		0.08*	
Negative consequences	0.33*		0.35*	
Social problems				
Step 1:Age & sex		0.02		0.02
Step 2		0.07		0.12
Screen time	0.03		0.02	
Heavy involvement	0.00		0.04	
Negative consequences	0.22*		0.29*	
Sleep problems				
Step 1:Age & sex		0.02		0.02
Step 2		0.06		0.06
Screen time	0.04		0.08*	
Heavy involvement	−0.02		0.05	
Negative consequences	0.18*		0.14*	
Sleep time				
Step 1:Age & sex		0.01		0.01
Step 2		0.02		0.02
Screen time	−0.09*		−0.08*	
Heavy involvement	−0.01		−0.02	
Negative consequences	−0.03		−0.04	

*Holm–Bonferroni adjusted value of *p*.

## 4. Discussion

The most important findings of the present study were that screen time is more strongly associated with symptoms of heavy involvement compared to negative consequences, and that it was primarily symptoms of negative consequences, not screen time, that contribute to the explained variance in health outcomes, except for sleep. In addition, the results of the present study showed that screen time and addictive use were more strongly associated for gaming compared to social media. For both gaming and social media, a substantial proportion of those meeting the full symptom criteria for IGD or SMD had relatively low levels of screen time and vice versa, suggesting that screen time is not a good diagnostic marker for either IGD or SMD.

### 4.1. Associations to negative health outcomes

Regarding associations with health outcomes, our finding of independent effects of addictive use, but not screen time, for most health outcome measures suggest that it is primarily addictive use of digital media that is related to health outcomes. Thus, the significant associations between screen time and health outcomes found in some previous studies [e.g., meta-analysis by Santos et al., 2023] might be misleading as they failed to control for to what extent the use is addictive or not.

The fact that we distinguished between two aspects of addictive use – heavy involvement and negative consequences – resulted in some important findings. In the regression analyses, negative consequences were associated with all health outcomes for both gaming and social media, except for sleep time. However, heavy involvement in gaming was only significantly associated with psychological problems, and heavy involvement in social media was only associated with low self-concept. Interestingly, the positive associations between heavy involvement and psychological problems changed to being negative in the regression analyses when controlling for the effect of screen time and negative consequences. This suggest that high levels of heavy involvement in gaming is associated with lower levels of psychological problems, but only if the gaming does not have negative consequences. This was, however, not the case for social media where high levels of heavy involvement continued to be positively associated with low self-concept in the regression analyses. The fact that high levels of heavy involvement in gaming, under some circumstances, are associated with lower psychological problems could be taken to be in line with the studies that have argued that highly-engaged, passionate gaming should not necessarily be regarded as addictive, even if this includes symptoms of preoccupation, withdrawal, and tolerance (e.g., Griffiths et al., 2016; Castro-Calvo et al., 2021; Infanti et al., 2023).

The only outcomes for which screen time had an effect beyond the influence of addictive use was for sleep. The independent effect of screen time for both gaming and social media on sleep time could be taken as support for displacement effects (Twenge, 2019), suggesting that a large amount of screen time crowds out sleep. We also found that screen time and symptoms of negative consequences of social media were independently related to sleep problems. This finding is interesting and probably relates to the fact that sleep problems can include both too little sleep and sleep of poor quality. A high screen time can result in a delayed bedtime, whereas symptoms of negative consequences from social media can result in low quality sleep, due to for example problems with friends and family as a result of problematic digital media use. The fact that only screen time for social media, and not screen time for gaming, had an independent effect for sleep problems could be related to the fact that it is relatively common to check social media at nighttime, which could lead to both short sleep time and low-quality sleep (Tandon et al., 2020). Finally, it should be noted that the sample size of the present study was large enough to detect even small effects, and screen time and addictive use for both gaming and social media explained only a small proportion of the variance in both sleep time and sleep problems.

Another finding worth mentioning is that the effects of screen time and addictive use of social media on psychological problems, low self-concept, and social problems were larger than the effects of gaming. These findings are in line with some previous research (Burén et al., 2021; Twenge and Farley, 2021) and could be taken as support for the notion that social media use can have serious negative consequences and that SMD, similarly to IGD, should be recognized as a diagnosis within the official classification systems, such as the DSM-5.

### 4.2. Associations of screen time and addictive use

In line with previous research (e.g., Wichstrøm et al., 2019; Burén et al., 2021), we made a distinction between IGD/SMD symptoms related to heavy involvement and those related to negative consequences. Interestingly, a discernible pattern emerged in the present study, indicating that symptoms related to heavy involvement (e.g., preoccupation, withdrawal, and tolerance) demonstrated stronger associations with screen time compared to symptoms indicating negative consequences (e.g., loss of interest, deception, and jeopardizing career/relationships). The exception was the symptom Escape, a symptom of negative consequences, which showed a strong association to screen time for gaming. As argued by for example Merhi (2016), playing games as an escape can induce positive feelings, which in turn is related to greater intention to play and a higher screen time. However, it has also been shown that at least from a more long-term perspective, the negative aspects of using games as an escape outweighs the positive ones (Hussain et al., 2021).

Another finding was that screen time and addictive use were more strongly associated for gaming than for social media. However, it should be noted that the association between screen time and addictive use was relatively strong also for social media. Stronger associations for gaming compared to social media was also found in one previous study (Bouna-Pyrrou et al., 2018). In addition, the present study provides new information by showing that associations are significantly stronger for gaming compared to social media also when investigating each one of the nine symptom criteria for IGD/SMD separately, except for the criterion jeopardizing career/relationships. These results could indicate that there are differences in the development of addictive use of different digital media. For example, individuals may have different motivations for using computer games and social media. Gaming is often used as a leisure activity (Hamari and Keronen, 2017), but also to boost one’s self-image by being recognized as a skillful player and receiving rewards (King and Delfabbro, 2009). To achieve these goals, considerable time spent gaming is required, which may explain why screen time for gaming is strongly associated with addictive use. Compared to gaming, social media include a wider range of content that users can get exposed to. When the social media contents are positive (e.g., body-positive content; Vandenbosch et al., 2022), spending time on social media may not be problematic. The results presented in Figure 1B can be taken as support for this, as it shows that a large proportion of those with no or only one SMD criteria has a high screen time for social media. However, if the contents are negative (e.g., appearance-based content and cyberbullying; Holland and Tiggemann, 2016; Kwan et al., 2020), even shorter amount of screen time can be problematic. For example, negative interactions on social media (e.g., being bullied, sexually harassed, or threatened) can create compulsive/addiction like behaviors to keep track of the situation without necessarily spending lots of time on social media. However, as the risk of being exposed to negative (and addictive) content increases with increased screen time, the effect of time is still relevant to disentangle from addictive symptoms. Conclusively, whereas high screen time for gaming often is associated with addictive use, the effects of extensive social media use may depend more on the content that one is exposed to.

Finally, we also found that a large proportion of those meeting the full symptom criteria for either IGD (35.3%) or SMD (31.5%) played for less than 30 h/week. Conversely, a large proportion of the participants who did not meet the criteria for IGD (45.8%) or SMD (48.6%) used digital media for more than 30 h/week, with some far exceeding this. These findings do not support the statement in DSM-5 (American Psychiatric Association, 2013) that individuals with IGD typically spend at least 30 h/week gaming, and further illustrates that spending large amounts of time gaming or using social media is not necessarily associated with addictive use and vice versa. As argued by, for example Pontes et al. (2022), focusing only on screen time therefore increases the risk of over-diagnosing IGD, and we show that this also applies for SMD. However, a focus on screen time can also result in an under-diagnosis among those with addictive use in combination with low screen time.

### 4.3. Limitations

Regarding limitations, the utilization of a cross-sectional design prevented causal inferences. Second, our reliance on self-report measures might have led to an overestimation of the associations due to shared-method variance and response biases. Using other reports to assess negative psychosocial outcomes would have been an advantage. As for addictive use, previous studies have shown discrepancies between logged measures and self-ratings of screen time (e.g., Parry et al., 2021), and associations between logged screen time and addictive use of gaming use has been found to be weak (Tomczyk and Lizde, 2023). However, logged measures of screen time also have limitations as screen time can be incorrectly logged if for example listening to music on your mobile while running. Thus, using a combination of different measures to assess addictive use would be recommended for future research.

## 5. Conclusion

Conclusively, the results of the present study showed significant associations between screen time and addictive use for both gaming and social media, with associations to screen time being significantly stronger for symptoms of heavy involvement compared to symptoms of negative consequences. However, when going beyond associations at the group level, it is evident that a large proportion of those with a high screen time for either gaming or social media meet no or only a few symptom criteria for IGD or SMD. This is in line with previous studies (e.g., Pontes et al., 2022), showing that screen time is a poor indicator of addictive use. More importantly, it was primarily the IGD/SMD symptom criteria related to negative consequences that contributed to most health outcomes when controlling for the overlap between screen time and the two types of addictive use. In fact, high levels of heavy involvement in gaming were even related to low levels of psychological problems when controlling for the overlap between the two aspects of addictive use and screen time. These findings emphasize the need to distinguish between digital media use that is passionate and highly-engaged versus use that is addictive (Griffiths et al., 2016; Castro-Calvo et al., 2021; Infanti et al., 2023), and also indicate that symptoms of negative consequences generally have a higher diagnostic validity compared to symptoms of heavy involvement (e.g., Burén et al., 2021; Castro-Calvo et al., 2021). It will be important for future research to further investigate the validity of each one of the nine symptoms for IGD that are included in the DSM-5 (American Psychiatric Association, 2013) and which symptoms that should be included if introducing SMD within diagnostical classification systems. Other factors that need to be explored more in detail in future research, and which could potentially be of importance for how to best individualize treatment and support, includes how different types of digital media content are associated with addictive use and mental health problems (e.g., Holland and Tiggemann, 2016; Kwan et al., 2020; Vandenbosch et al., 2022) and what motives that an individual has for using digital media (e.g., Kircaburun et al., 2020; Bäcklund et al., 2022).

## Data availability statement

The raw data supporting the conclusions of this article will be made available by the authors, without undue reservation.

## Ethics statement

Ethical approval was not required for the studies involving humans because according to the national-legislation Swedish Act 2003:460, a formal ethical application is not required for anonymous survey studies on participants aged 15 or above, and that do not include serious risks. Both adult and underage participants were required to provide written informed consent. Written informed consent from the underage participants’ legal guardians/next of kin was not required as the underage participants were 15 years or older (in line with the national legislation-Swedish Act 2003:460). The studies were conducted in accordance with the local legislation and institutional requirements. The participants provided their written informed consent to participate in this study.

## Author contributions

JB: Conceptualization, Data curation, Formal analysis, Investigation, Methodology, Project administration, Writing – original draft, Writing – review & editing. SN: Conceptualization, Investigation, Methodology, Writing – review & editing. LT: Conceptualization, Formal analysis, Funding acquisition, Investigation, Methodology, Project administration, Resources, Supervision, Writing – review & editing.
